# Longitudinal bioluminescence imaging to monitor breast tumor growth and treatment response using the chick chorioallantoic membrane model

**DOI:** 10.1038/s41598-022-20854-9

**Published:** 2022-10-13

**Authors:** Sumreen Javed, Sepideh Soukhtehzari, Nazarine Fernandes, Karla C. Williams

**Affiliations:** Faculty of Pharmaceutical Sciences, The University of British Columbia, Vancouver, Canada

**Keywords:** Cancer, Breast cancer, Tumour angiogenesis

## Abstract

The development of successful treatment regimens for breast cancer requires strong pre-clinical data generated in physiologically relevant pre-clinical models. Here we report the development of the chick embryo chorioallantoic membrane (CAM) model to study tumor growth and angiogenesis using breast cancer cell lines. MDA-MB-231 and MCF7 tumor cell lines were engrafted onto the chick embryo CAM to study tumor growth and treatment response. Tumor growth was evaluated through bioluminescence imaging and a significant increase in tumor size and vascularization was found over a 9-day period. We then evaluated the impact of anti-angiogenic drugs, axitinib and bevacizumab, on tumor growth and angiogenesis. Drug treatment significantly reduced tumor vascularization and size. Overall, our findings demonstrate that the chick embryo CAM is a clinically relevant model to monitor therapeutic response in breast cancer and can be used as a platform for drug screening to evaluate not only gross changes in tumor burden but physiological processes such as angiogenesis.

## Introduction

Breast cancer is a heterogeneous disease that presents with diverse clinical features and extensive genetic and epigenetic variations. The development of new therapeutics with utility in breast cancer treatment relies heavily on in vitro cell-based models as a testing platform for evaluating tumor response before transitioning into animal models for validation studies. A common pre-clinical approach for studying therapeutic targets in breast cancer is through the use of 2D and 3D in vitro cell culture-based models^1–3^. 2D cell culture assays involve seeding cells on a plastic dish, while useful, and necessary, for assays such as target-engagement this method does not account for the complex interactions of tumor cells with the extracellular matrix. To mimic the tumor-ECM interaction, 3D cell culture can be employed^4,5^. Successful results in a 2D or 3D setting provide rational for testing therapeutics in an in vivo setting, generally using the mouse or rat model^6^. All of these models can aid in enhancing our understanding of tumor growth, metastasis, and drug resistance. However, successful results in a rodent model are generally required for the implementation of a therapeutic into a clinical trial.

The use of animals in research is restricted by the application of 3Rs namely: reduction, refinement, and replacement of the animal. In addition to being costly and time consuming, there are limitations to the testing of multiple drug formulations or drug screening^7^. This highlights the need for an intermediate model that can mimic an in vivo setting by generating a complex 3D environment supporting tumor cell growth and vascularization while being amenable to larger drug screens. A model with great potential to aid in improving our understanding of cancer development and support precision medicine efforts is the chick embryo model. The chick embryo chorioallantoic membrane (CAM) model has been highlighted as an excellent in vivo model for studying cancer progression in real-time^8–10^. Chief among the advantages of this model is the ease of tumor engraftment without rejection due to an immunodeficient state. Additionally, the presence of highly organized capillary bed networks provided by the CAM supports tumor vascularization and is critical for studies assessing pro and anti-angiogenic factors that might affect tumor growth and progression. Of practical consideration, the low cost and economic feasibility of the model has the potential to support large-scale drug screen studies and, potentially, personalized medicine using patient-derived xenografts (PDXs)^7–11^.

Tumor angiogenesis is a classic hallmark of cancer and in vitro and in vivo studies focused on elucidating the mechanisms of tumor vascularization have led to the development of anti-angiogenic drugs targeting VEGF signaling pathways such as: bevacizumab, axitinib, and sunitinib^4,12^. Preclinical studies investigating tumor cell response to bevacizumab demonstrated a significant reduction in tumor burden^13^. This lead to the approval of bevacizumab for the treatment of breast cancer which remains in place in Europe. Another anti-angiogenic drug with demonstrated utility in pre-clinical models axitinib. Axitinib has been approved for renal cell carcinoma and may have potential utility in other cancer types^26^.

Here, we have optimized the chick embryo CAM model for the engraftment of breast cancer cell line-derived tumor spheres to recapitulate tumor growth and angiogenesis. We then tested the anti-angiogenic drugs, axitinib and bevacizumab, and evaluated tumor response to treatment. Our results detail the successful engraftment and monitoring of breast tumor growth using the chick embryo CAM model and highlight this model as a platform to evaluate the effects of a therapeutic on tumor growth and angiogenesis.

## Methods

### Cell lines and cell culture

The triple negative breast cancer cells MDA-MB-231 (ATCC HTB-26) and estrogen positive breast cancer cells MCF-7 (ATCC HTB-22) were obtained from the ATCC and cultivated in DMEM with 10% fetal bovine serum (FBS; Gibco Life Technologies, Grand Island, NY, USA). MCF-10A (CRL-10317), was obtained from ATCC and cultivated in DMEM with horse serum (5%), EGF (20 ng/mL), insulin (10 µg/ml), hydrocortisone (0.5 mg/mL), and cholera toxin (100 ng/mL). These cells were cultured at 37 °C in humidified environment and were supplemented with 5% CO_2_. For all experiments cells were between 70 and 80% confluent at the time of experiment initiation and cell viability, assessed by trypan blue, was greater than 90%. All cell lines were bio-engineered to express firefly luciferase using lentivirus transduction. Neomycin-resistant bioluminescent plasmid expressing firefly luciferase was purchased from Addgene (Plasmid #21471)^14^. For the in-house virus particle formation, HEK293 T cells were transfected with turbofectin, plasmid, gag, and env genes. After 24 and 48 h of incubation, virus particle-rich media was collected, centrifuged, and stored in −80 °C. On the day of infection, the cells were treated with the virus particles and polybrene (Sigma-Aldrich TR-1003-G). Luciferase positive cells were selected using neomycin, G418 (700 µg/mL). Confirmation of luciferase expression was determined through bioluminescence imaging using an IVIS Lumina (Caliper Lifesciences, Waltham, MA).

### 3-D tumor sphere formation

Tumor spheres were prepared two days prior to xenografting onto the chick embryo CAM. Cell number per sphere was optimized for each cell line based on measured BLI tumor growth over a 9-day period (Supplemental Fig. 1A–H). 2.5 × 10^5^ cells per sphere for MDA-MB-231 cells and 5 × 10^4^ cells per sphere for MCF7 were used for all experiments. Cells were resuspended in 50µL of Matrigel® and seeded as a droplet on a 10 cm culture plate. Cell/matrigel spheres were incubated for half an hour at 37 °C to solidify and then incubated for 2 days under standard cell culture conditions.

### Ex ovo chicken chorioallantoic membrane assay

Fertilized eggs were obtained from Poultry Research Centre (AB, Canada) and incubated at 37 °C with rotation for three days. On embryonic development day (EDD) three, eggs were cracked as previously described^10^. Tumor xenografts were performed on EDD nine. Xenografting was performed using breast cell line-derived spheres. Before onplanting, spheres were washed with PBS (once) and the baseline BLI was measured. For BLI measurements, ~ 20µL luciferin (15 mg/mL) was topically added onto spheroids, incubated for 5 min, and imaged on an IVIS Lumina (Caliper Lifesciences, Waltham, MA) using the BLI optical imaging setting for 10 s. The sphere was placed onto a scored area of the CAM. The growth of the tumor was monitored on Day 2, 4, 7 and 9 post-engrafting using IVIS imaging. At end-point, Day 9 post-engraftment, the tumors were resected, fixed and processed for immunohistochemistry.

### Antiangiogenic drug testing

Axitinib was purchased from SelleckChem (Houston, TX). Axitinib was diluted in DMSO and stored at −20 °C at a stock concentration of 10 mM. Chick embryo tumor sphere xenografts were treated topically with either 30µL axitinib (10 µM) or control (DMSO) on Day 4 and Day 7 post-engraftment. Tumor growth was monitored using IVIS imaging and at end-point, Day 9 post-engraftment, tumors were resected, fixed and processed for immunohistochemistry. Similarly, Bevacizumab was also purchased from SelleckChem (Houston, TX). Chicken embryo spheres were treated with 10.5µL of Bevacizumab (7.5 mg/kg) or control on Day 4 and 7 post engraftment and tumor growth was monitored through IVIS.

### Immunohistochemistry

Hematoxylin and Eosin (H&E) staining was performed for histological analysis of the tumor tissues. Tumors grown on the chick CAM were removed, fixed in 4% PFA, stored in ethanol, followed by paraffin-embedding. Embedded tumors were sectioned at 5 μm, baked overnight at 37 °C, followed by deparaffinized with xylene and rehydration using an ethanol gradient (100%, 95%, 70%, 50%) followed by a 1X phosphate-buffered saline (PBS) wash. For H&E staining, sections were then stained with Hematoxyline and Eosin kit as per manufacturer instruction (Vector Labs, Cat No H-3502). For Ki67 immunostaining, tissue sections were deparaffinized and dehydrated as mentioned above. Then heat-induced antigen retrieval was performed at 95 °C for 40 min in 10 mmol/L citrate buffer (pH 6). After heating, slides were cooled to room temperature and washed in 1X PBS. Sections were permeabilized in 0.1% Triton X-100 in PBS and then blocked for 20 min in 10% goat serum in PBS for 20 min. (Vector Labs, Cat No. S-1000). Slides were washed with PBS and incubated with Ki67 rabbit polyclonal antibody at 1:1000 (Abcam, Cat No. ab15580) for 1 h at room temperature. Excess antibodies were removed by washing three times with 1XPBS. Slides were incubated for 1 h at room temperature with a biotinylated goat anti-Rabbit IgG (1:200, Vector Labs), washed with PBS, followed by horseradish peroxidase (HRP) streptavidin for 30 min at room temperature and the stained with DAB substrate (3–3’-Diaminobenzidine) (Vector Labs, SK-4100). Slides were counterstained with Harris hematoxylin, dehydrated and mounted with Vecta mount medium (Vector Labs).

### Data analysis

Data was collected, handled in Excel, and analyzed using GraphPad Prism 9.1.2. Data passing the Kolmogorov–Smirnov normaility test was analyzed by a Student’s two-tailed unpaired T-test or ordinary one-way ANOVA Tukey’s multiple comparison test. If normality failed, data was analyzed using non-parametric Mann–Whitney test or Kruskal–Wallis ANOVA test. Data was normalized to the BLI reading from each individual tumor on D0. Angiogenesis analysis was performed using ImageJ® software. The raw image was imported into ImageJ and converted into an 8-bit image. The draw ellipse function was used to position an ellipse over the entire tumor area. The ellipse was then subtracted using the ‘clear’ function to remove the tumor sphere. The image ‘Threshold’ was set to default (black and white) and adjusted. An ellipse area of x = 200 and y = 200 pixels surrounding the subtracted tumor was selected and pixels outside the 200 × 200 region were where removed using the ‘clear’ function. Pixels in the 200 × 200 region represented vasculature penetrating the tumor. ‘Particle Analysis’ function was performed on this area so that the vessels surrounding the tumor were quantified (total pixels). All data presented are from an average of at least three independent experiments, unless otherwise stated. Statistical tests used are noted in the figure legend. Error bars represent the standard error of the mean (SEM). Statistical significance of different groups was determined by a* p*-value under 0.05 and denoted accordingly. Graphs were prepared in GraphPad Prism and figures were prepared in Adobe Illustrator CC 2022.

### Ethics approval

All experimental protocols carried out in this study were in accordance with institution protocol and study approval was obtained by the institutional biosafety review committee of UBC (BIO#B17-0151). The study was carried out in compliance with the ARRIVE guidelines.

## Results

### Generation of bioluminescent tumor spheres for modelling tumor growth

**Figure 1:**
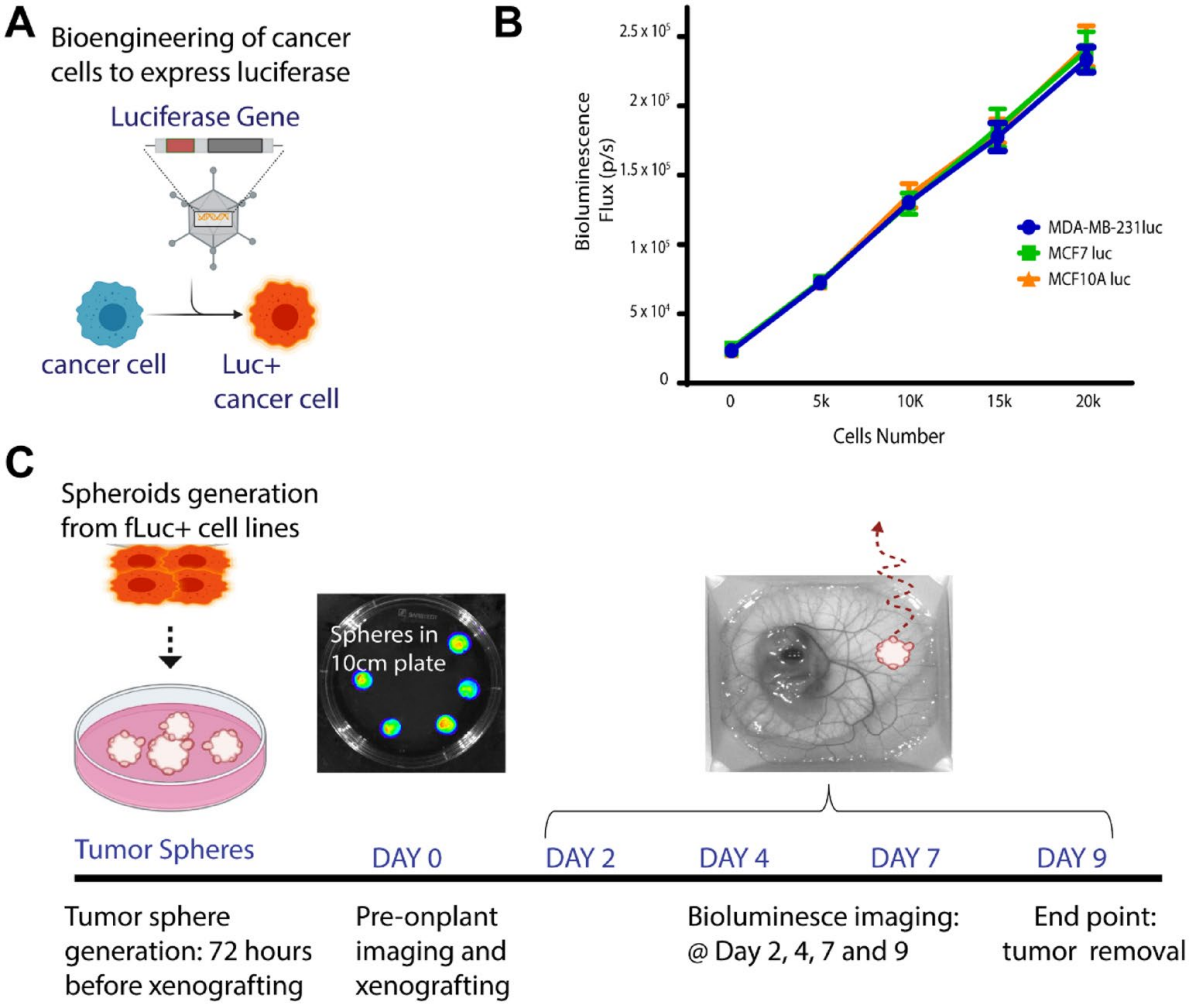
Method to study breast tumor growth using the chick embryo model and longitudinal bioluminescence imaging. (**A**) Generation of firefly luciferase expressing cell lines and (**B**) their relative bioluminescence using an increasing cell number for each cell line: MDA231, MCF7, MCF10A lines (5 k, 10 k, 15 k, and 20 k). (**C**) Schematic diagram representing the experimental timeline from spheroid generation, chick CAM BLI and tumor removal.

Bioluminescence imaging using luciferase reports, such as firefly luciferase, permits longitudinal studies to monitor tumor growth. An advantage of firefly luciferase (fLuc) is the enzymes requirement for ATP for activity, meaning that only viable cells are capable of cleaving luciferin and producing bioluminescence^15^. To generate cell lines expressing firefly luciferase, we packaged a firefly luciferase plasmid into lentivirus and performed lentiviral infection using MDA-MB-231, MCF-7, and MCF10A cell lines (Fig. 1A). We validated luciferase expression for each cell line using bioluminescence imaging (BLI) and cell number titrations. All three cell lines were found to exhibit similar fLuc expression as assessed by BLI (Fig. 1B). We then developed a system to generate tumor spheres and monitor tumor growth rates using the chick embryo CAM. Tumor spheres were generated by resuspending cell lines in Matrigel which were dropped onto a cell culture dish, polymerized at 37 °C, and grown in standard cell culture conditions for 48 h. Prior to engraftment onto the chick embryo CAM, tumor spheres were imaged by BLI. Tumors were then engrafted on the chick embryo CAM and tumor growth was monitored by BLI every 2–3 days (Fig. 1C).

### Tumor engraftment of breast cell lines onto the chick embryo chorioallantoic membrane and monitoring of tumor growth rates

**Figure 2:**
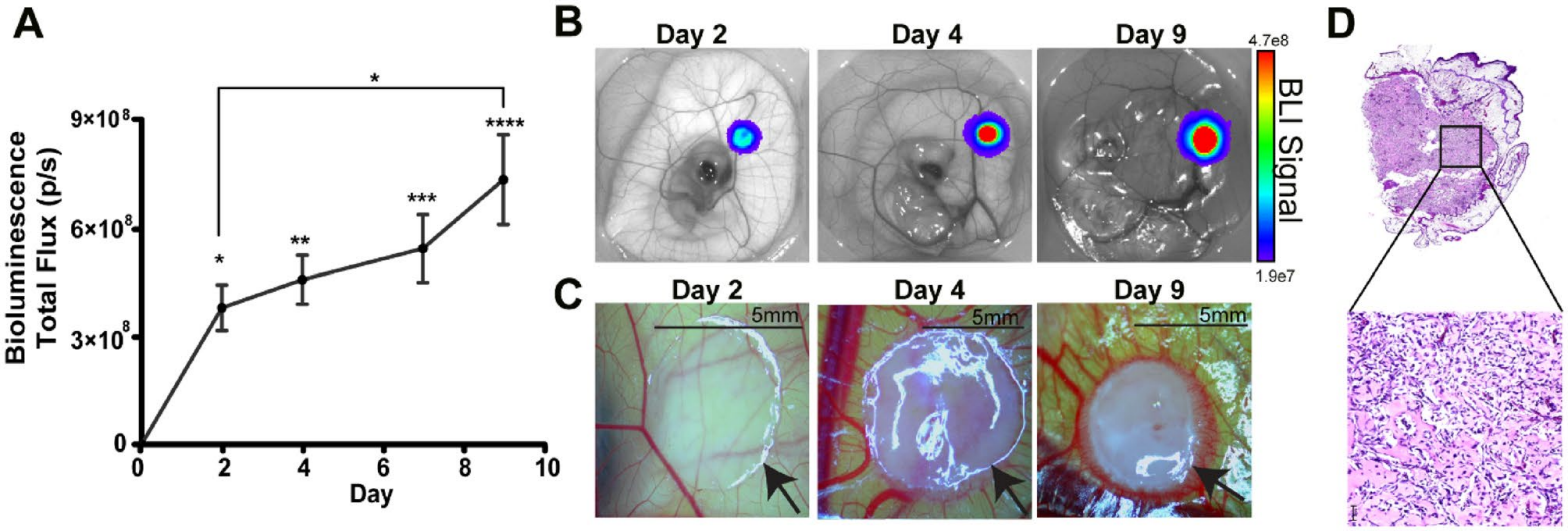
Engineered MDA-MB-231luc spheroids growth profile on the chick embryo chorioallantoic membrane (CAM). (**A**) Tumor spheroid growth curve of engineered MDA-MB-231luc spheroid (2.5 × 10^5^ cells/spheroid) on CAM post engrafting (n = 16). Error bars represents SEM, asterisk denotes significance, Tukey’s test. (**B**) Representative image of MDA-MB-231luc spheroid on CAM measured via IVIS. (**C**) Representative image of MDA-MB-231luc spheroid on CAM observed via light microscope. (**D**) Representative image of H/E staining of MDA-MB-231luc spheroid on CAM.

Tumor spheres were generated using MDA-MB-231, MCF-7, and MCF-10A cell lines. On day 0 (D0) spheroids were imaged and engrafted on the chick embryo CAM. Tumor growth was then monitored using BLI every 2–3 days. Optimization of cell number per tumor sphere was performed by examining tumor sphere growth over a 9-day period, as assessed by BLI (Supplemental Fig. 1A–H). MDA-MB-231luc spheres (0.25 × 10^6^ cells/sphere) demonstrated significant increases in tumor size over time from D0 (Fig. 2A and B). In addition, a significant increase in tumor size between D2 and D9 was also identified (Fig. 2A). Observed changes in tumor size and vascularization was also found and, visually, tumors increased in size and vascularization surrounding the tumor was apparent (Fig. 2C and D).

**Figure 3:**
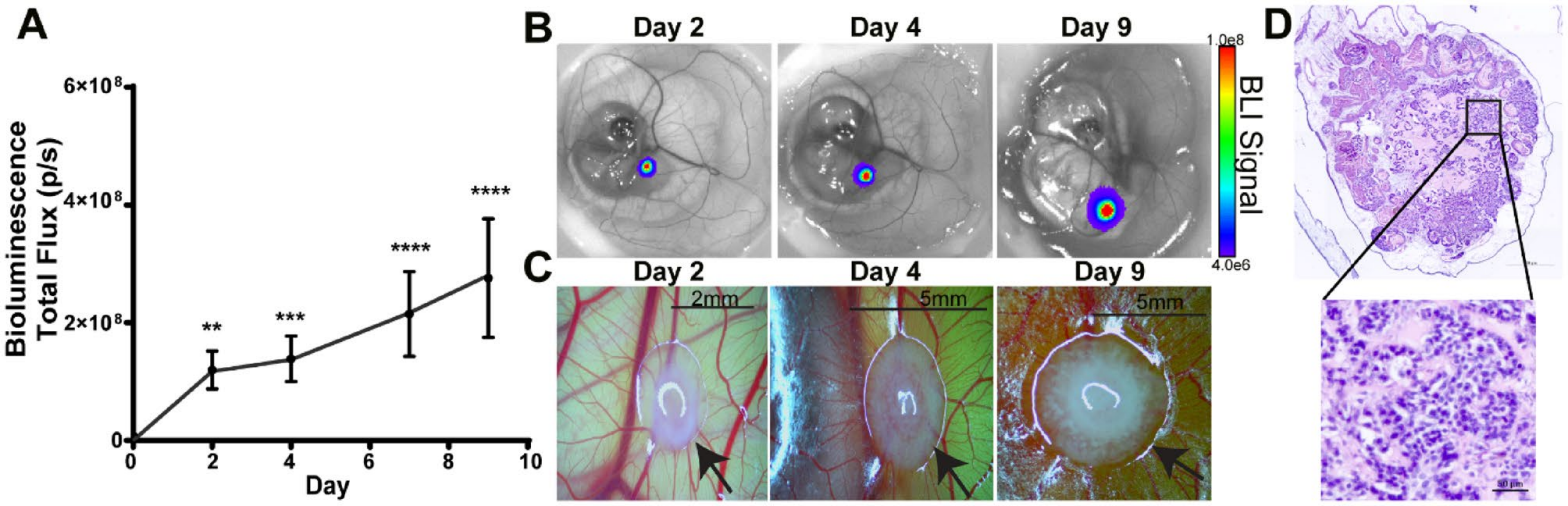
Engineered MCF7luc spheroids growth profile on the chick embryo chorioallantoic membrane (CAM). (**A**) Tumor spheroid growth curve of engineered MCF7luc spheroid (0.5 × 10^5^ cells/spheroid) on the chick CAM post engrafting (n = 14). Error bars represent SEM, asterisk denotes significance, Kruskal–Wallis test. (**B**) Representative image of MCF7luc spheroid on CAM measured via IVIS. (**C**) Representative image of MCF7luc spheroid on CAM observed via light microscope. (**D**) Representative image of H/E staining of MCF7luc spheroid on CAM.

Next, we assessed if a hormone-receptor positive breast tumor cell line would grow on the chick embryo CAM. MCF7luc spheres were engrafted on surface of the CAM and tumor growth was monitored by BLI. Tumors (0.5 × 10^5^ cells/spheres) were found to demonstrate significant growth over time from D0 (Fig. 3A and B). Observed changes in tumor size and vascularization was also found and, visually, tumors increased in size and vascularization surrounding the tumor was apparent (Fig. 3C and D). Tumor volume was also manually measured for both MDA-MB-231luc and MCF7luc tumors. MDA-MB-231luc tumors showed an increase in tumor volume between D2 and D7; however, D9 did not demonstrate an increase in tumor volume and a reduction in size was found (Supplemental Fig. 1I). MCF7luc tumor volume was manually measured and a small increase in tumor volume was found (Supplemental Fig. 1J). As we observed that tumor growth can occur on the underside of the CAM, which cannot be effectively measured through a top-surface measurement, estimating tumor volume on the CAM without removal of the tumor may underestimate tumor size. This suggests that BLI is a more accurate way to monitor tumor growth in the CAM model.

**Figure 4:**
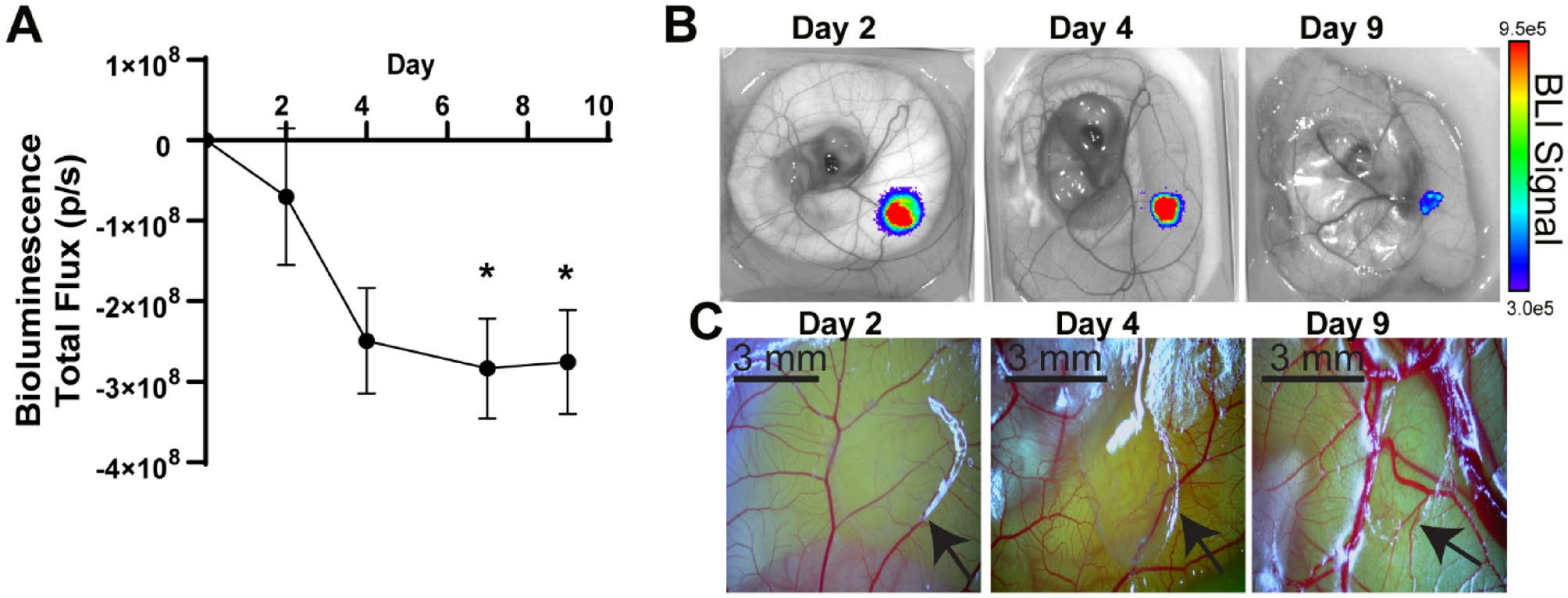
Engineered MCF10Aluc spheroids growth profile on the chick embryo chorioallantoic membrane (CAM). (**A**) Tumor spheroid growth curve of engineered MCF10Aluc spheroid on CAM post engrafting (5 × 10^4^ cells/spheroid N = 4). Error bar represents SEM, asterisk denotes significance, Tukey’s test. (**B**) Representative image of MCF10Aluc spheroid on CAM measured via IVIS. (**C**) Representative image of MCF10Aluc spheroid on CAM observed via light microscope.

Our results demonstrate that breast tumor growth can be monitored using BLI of tumors engrafted onto the chick embryo CAM. Next, we assessed if a non-tumorigenic breast cell line would display similar growth characteristics. MCF10Aluc spheres were xenografted onto the CAM surface and monitored for growth using BLI. MCF10Aluc spheres did not exhibit a growth profile and rather showed a significant reduction in bioluminescence over time (Fig. 4A and B). There was no observed tumor growth or changes in vascularization to the area where the spheroid was engrafted (Fig. 4C). Modifying the number of cells engrafted did not alter the growth profile and a significant decrease was found for all conditions (Supplemental Fig. 1F–H).

### Effects of axitinib on tumor growth and angiogenesis

**Figure 5:**
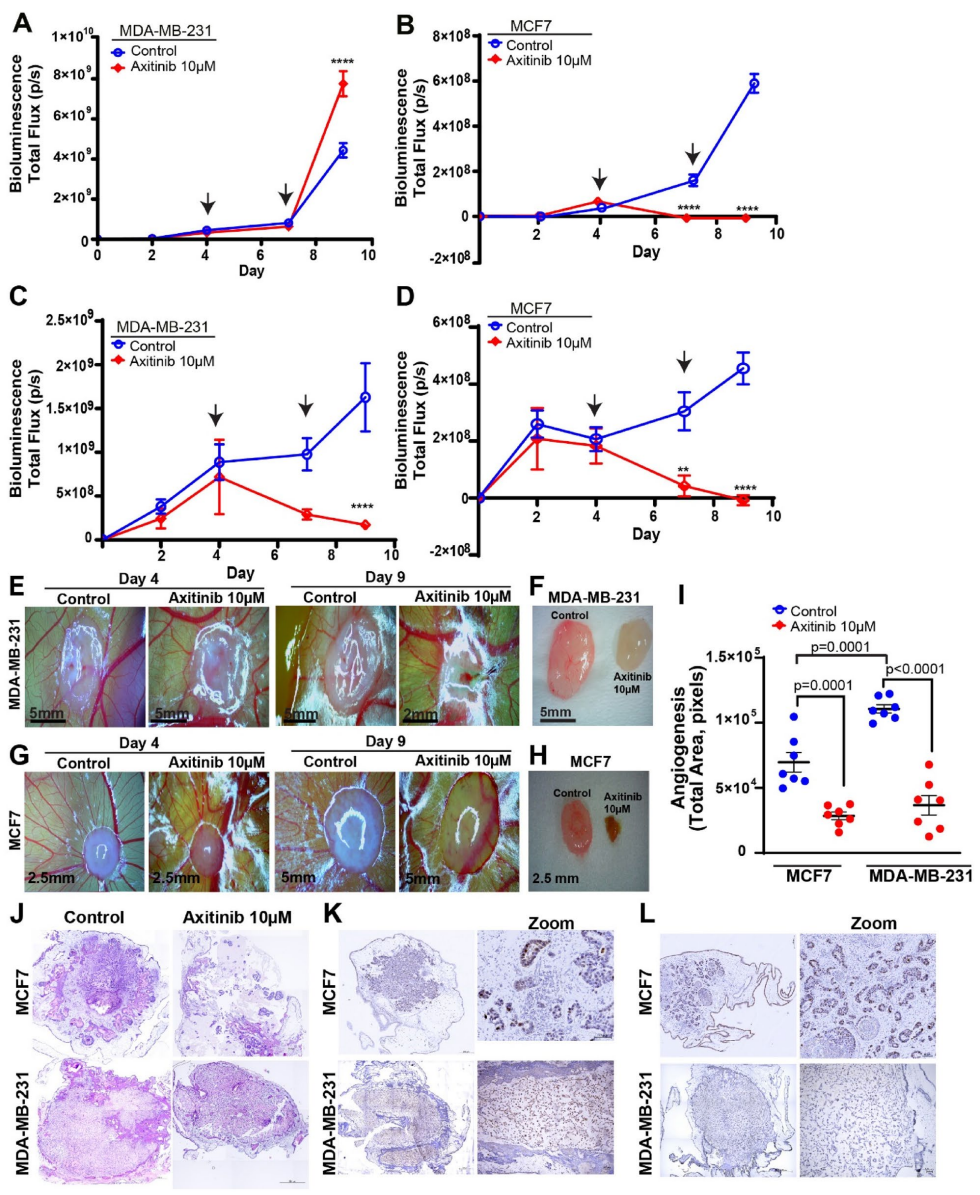
Axitinib treatment in MDA-MB-231luc and MCF7luc spheres. (**A**,** B**) Axitinib treatment of (**A**) MDA-MB-231luc and (**B**) MCF7luc tumor spheres in vitro (n = 10 per group). (**C**, **D**) Axitinib treatment of (**C**) MDA-MB-231luc and (**D**) MCF7luc tumor spheres in vivo, xenografted onto the chick embryo CAM (n = 7–10 per group). Error bars in both graphs represents SEM, asterisk denotes significance, Two-way Anova Multiple Comparison Test. Black arrows demonstrate the days of the treatment (Day 4 and Day 7). (**E**, **G**) Representative image of treated, or untreated, tumors on Day 4 and Day 9 for (**E**) MDA-MB-231luc cells and (**G**) MCF7luc cells when observed via a light microscope. (**F**, **H**) Comparison of control (left) and treatment (right) tumor size when resected at endpoint (Day 9) for (**F**) MDA-MB-231luc cells and (**H**) MCF7luc. (**I**) Total angiogenic area in the tumor periphery of MDA-MB-231 and MCF7 tumors spheres xenograft onto the chick embryo CAM at endpoint (Day 9) treated with axitinib or DMSO control (n = 7 per group). Error bars represents SEM, * p*-value denotes significance, Tukey’s test. (**J**) Representative H/E staining of MDA-MB-231luc and MCF7luc control and axitinib-treated tumors harvested from the CAM. (**K**,** L**) Representative Ki67 staining of tumor sections (left row) and zoom insets (right row) for: (**K**) MDA-MB-231luc and MCF7luc control and (**L**) axitinib-treated tumors harvested from the CAM.

Having demonstrated the feasibility of the chick embryo model to monitor tumor growth and angiogenesis, we evaluated the capabilities of this model to monitor and quantify response to treatment. For this, we utilized a known anti-angiogenic agent axitinib and evaluated tumor growth and vascularization. First, we evaluated the effects of axitinib on MDA-MB-231luc and MCF7luc tumors spheres grown in vitro. Tumor spheres were grown and maintained in cell culture conditions for 9 days. Tumor spheres were treated with axitinib on Day 4 and 7 and monitored for tumor growth by BLI. MDA-MB-231luc control and axitinib spheres demonstrated similar growth profiles before and after the first treatment (Fig. 5A). However, after the second axitinib treatment a significant increase in tumor growth was found for the treatment group compared to the control (Fig. 5A). MCF7luc tumor spheres demonstrated a significant reduction in tumor growth following axitinib treatment compared to control (Fig. 5B).

To investigate the effect of axitinib on the growth of tumor sphere xenografts, we xenografted MDA-MB-231luc and MCF7luc spheres on the CAM and topically added axitinib on day 4 and 7. Tumor growth was monitored using BLI and a significant reduction in tumor size was found for MDA-MB-231luc and MCF7luc tumors (Fig. 5C and D). Tumors treated with axitinib were visibly smaller compared to control treated tumors as assessed during treatment (Fig. 5E and G) and at end-point when tumors were removed (Fig. 5F and H).

Next, we quantified tumor angiogenesis by measuring the vascular area surrounding the tumor. A significant reduction in tumor angiogenesis was found MDA-MB-231luc and MCF7luc tumors treated with axitinib compared to control (Fig. 5I). Interestingly, we also found significantly lower levels of vascularization for MCF7luc tumors compared to MDA-MB-231luc tumors (Fig. 5I). This suggests that MCF7 cells are less angiogenic compared to more invasive cell line MDA-MB-231. Histological assessment of control and axitinib treated tumors also showed a reduction in size for the axitinib treated tumors (Fig. 5J–L).

### Effects of bevacizumab on tumor growth and angiogenesis

**Figure 6:**
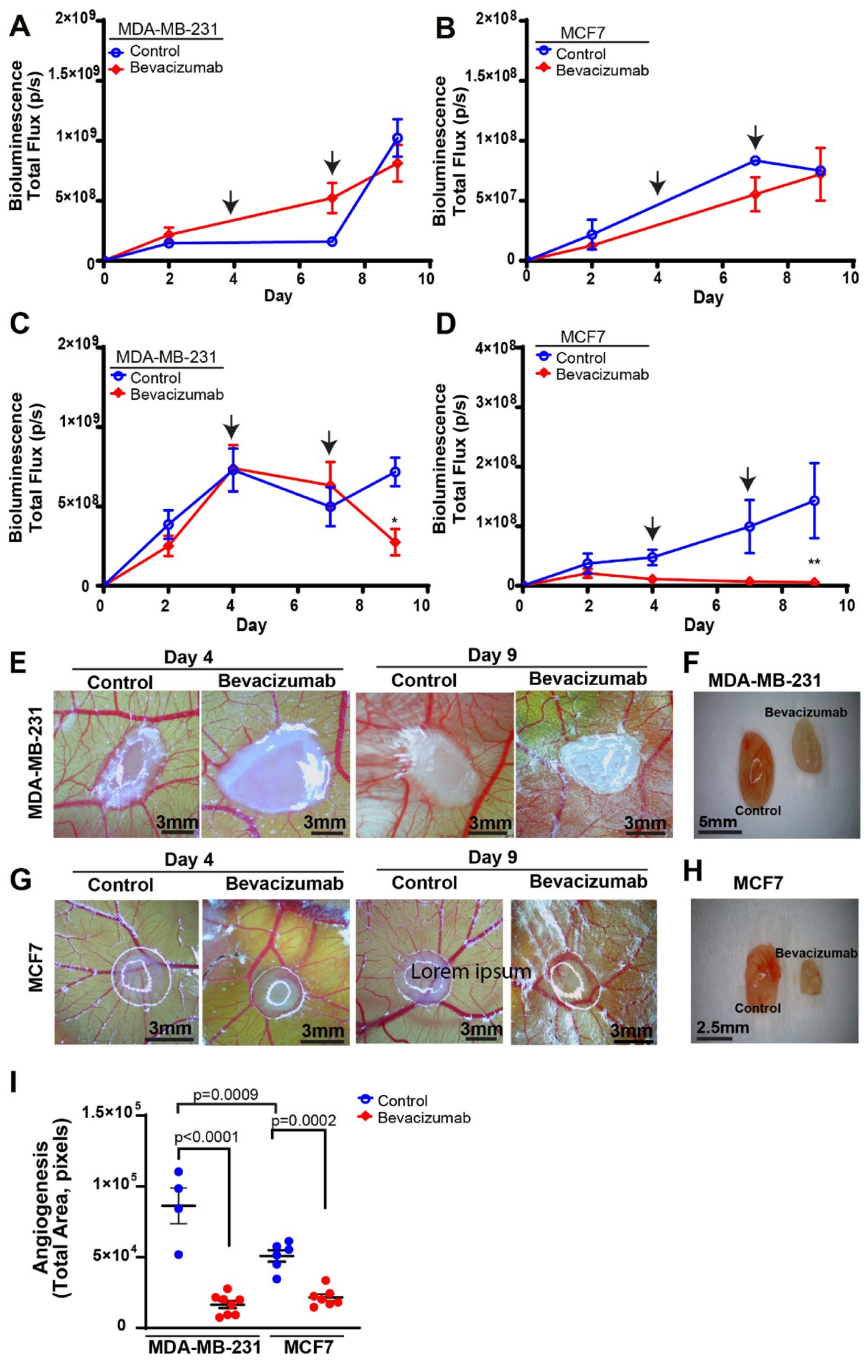
Bevacizumab treatment in MDA-MB-231luc and MCF7luc spheres. (A and B) Bevacizumab treatment of (**A**) MDA-MB-231luc and (**B**) MCF7luc tumor spheres in vitro (n = 5 per group). (**C**, **D**) Bevacizumab treatment of (**C**) MDA-MB-231luc and (**D**) MCF7luc tumor spheres in vivo, xenografted onto the chick embryo CAM (n = 5–7 per group). Error bars in both graphs represents SEM, asterisk denotes significance, Two-way Anova Multiple Comparison Test. Black arrows indicate the days of the treatment (Day 4 and Day 7). (**E**, **G**) Representative image of treated, or untreated, tumors on Day 4 and Day 9 for (**E**) MDA-MB-231luc cells and (**G**) MCF7luc cells when observed via a light microscope. (**F**, **H**) Comparison of control (left) and treatment (right) tumor size when resected at endpoint (Day 9) for (**F**) MDA-MB-231luc cells and (**H**) MCF7luc. (**I**) Total angiogenic area in the tumor periphery of MDA-MB-231 and MCF7 tumors spheres xenograft onto the chick embryo CAM at endpoint (Day 9) treated with axitinib or DMSO control (n = 7 per group). Error bars represents SEM, * p*-value denotes significance, Tukey’s test.

After establishing the utility of this model to test axitinib, a relatively newer antiangiogenic drug, we also tested a well-known antiangiogenic drug, bevacizumab, to evaluate its effect on tumor growth in vitro and in vivo. For this, we first evaluated the effects of bevacizumab on MDA-MB-231luc and MCF7luc tumors spheres grown in vitro. Tumor spheres were grown and maintained in cell culture conditions for 9 days. Tumor spheres, in vitro, were treated with bevacizumab on Day 4 and Day 7 and monitored for growth by BLI. MDA-MB-231luc and MCF7luc control and bevacizumab treated tumor spheres demonstrated similar growth profiles before and after treatment (Fig. 6A and B).

Next we investigated the effects of bevacizumab on tumor growth in vivo, xenografted onto the chick CAM. Tumor spheres xenografted onto the CAM were treated with bevacizumab on Day 4 and Day 7. Tumor response to treatment was monitored using BLI and a significant reduction in tumor size for MDA-MB-231luc and MCF7luc tumors was found on Day 9 (Fig. 6C and D). Tumors treated with bevacizumab were visibly smaller compared to control treated tumors post-treatment (Fig. 6E and G). This was also observed at end-point when tumors were removed (Fig. 6F and H). Next, we quantified tumor angiogenesis by measuring the vascular area surrounding the tumor. A significant reduction in tumor angiogenesis was found MDA-MB-231luc and MCF7luc tumors treated with bevacizumab compared to control (Fig. 6I). This demonstrates that bevacizumab is able to reduce tumor size, but only in vivo when angiogenesis is required to support growth. Overall, these results demonstrate the utility of the chick embryo CAM model as an effective and sensitive model to monitor in vivo tumor growth and response to treatment.

## Discussion

Our lab, and others, have used the chick CAM model for studying various aspects of cancer growth and metastasis^9,10,16–20^. Here we report the use of the chick embryo model to monitor tumor growth, vascularization, and response to treatment. Our work has led to the optimization of tumor growth, as monitored using BLI, for two breast cancer cell lines and demonstrates that the non-tumorigenic cell line, MCF10A, does not successfully grow on the chick embryo CAM. We demonstrate that this model can be used to assess physiologically important processes in tumor development, such as angiogenesis. The in vivo results from this model can then be compared to in vitro results, thereby supporting an assessment of drug-mediated autocrine versus paracrine effects.

To establish the use of CAM for the successful growth of the breast tumors, we used a triple negative cancer cell line, MDA-MB-231 and a hormone receptor positive cell line, MCF7. Our results show that MDA-MB-231 and MCF7 spheres grow successfully on CAM. We also found that MDA-MB-231 tumors exhibited more vascularization as compared to the MCF7 tumors which might corresponds to the aggressiveness of the tumor and its tendency to make neo-vasculatures^21^. We selected the anti-angiogenic agent axitinib to test in our model. Axitinib is newer anti-angiogenic agent with demonstrated safety and efficacy in renal cell carcinoma. Axitinib in combination with avelumab or pembrolizumab has demonstrated superiority over standard-of-care sunitinib in advanced renal cell carcinoma^22,23^. In addition, axitinib does not exhibit negative drug–drug interactions with standard chemotherapies and can be used as a combination treatment^24^. All of this highlights the demonstrated clinical utility of axitinib as a cancer therapeutic. We tested axitinib on tumor spheres in an in vitro setting and found that axitinib reduced tumor growth, but in only the MCF7 cell line. Axitinib did not reduce tumor sphere growth of MDA-MB-231, and we found a significant increase in tumor proliferation after the second drug treatment. However, in vivo, we found axitinib significantly reduced tumor growth and tumor vascularization. For MCF7 cells, axitinib was able to inhibit tumor growth in vitro as well as in vivo whereas for MDA-MB-231 cells only in vivo tumor growth was affected. This suggests that axitinib may exhibit a different mechanism of action for hormone positive and triple negative breast tumors. Next, we tested bevacizumab, a well-known antiangiogenic drug, to assess tumor sphere response to treatment in an in vivo and in vitro setting. Bevacizumab did not reduce MDA-MB-231 and MCF7 tumor spheroid growth in vitro, while in vivo bevacizumab significantly reduced tumor growth. This suggests that, in breast cancer, bevacizumabs primary mechanism of action is inhibition of tumor angiogenesis whereas axitinib may function to impair angiogenesis and, for hormone-positive breast cancer, directly reduce tumor cell proliferation.

Our work suggests several advantages of the CAM model for studying tumorigenesis. First, tumor growth can be evaluated using BLI which permits non-invasive and longitudinal monitoring of tumors. Secondly, tumor vascularization can be evaluated and histological assessment of tumors at end-point could be used to evaluate changes in cell phenotype. Third, this model can be employed to test different pharmaceutical agents, which due to its cost effectiveness and minimal handling requirement, would have utility in screening multiple compounds. This could permit the devaluation of multiple compounds and support the selection of key compounds, or combinations, for evaluation in the mouse model.

Our observations are consistent with the work performed by Jefferies et al.^25^ whose findings indicated that the CAM model can be employed to monitor the growth of human prostate cancer tumors and osteosarcoma via non-invasive BLI method. However, we were able to obtain consistently increasing values of the grafted tumor, which suggests successful tumor growth for nine days. We were also able to show the anti-angiogenic effect of axitinib which is supported by the work performed by Berndsen et al.^26^ in colorectal cancer cell lines. Overall, our findings strongly support the use of chick embryo CAM in evaluating tumor response to treatment which has important implications for drug screening in the in vivo setting.

## Supplementary Information

Supplementary Information.

## Data Availability

All data supporting the conclusions of this research is provided in this article and in supplemental files. All raw datasets generated during and/or analysed during the current study are available from the corresponding author on reasonable request.
