# Supplementary material for: Longitudinal bioluminescence imaging to monitor breast tumor growth and treatment response using the chick chorioallantoic membrane model
Source: Sci Rep. 2022 Oct 13;12:17192. doi: 10.1038/s41598-022-20854-9 (PMC9562337; doi:10.1038/s41598-022-20854-9)
Supplement: Supplementary file 1 — Supplementary Information. [file 41598_2022_20854_MOESM1_ESM.docx]

**SUPPLEMENTAL FIGURES**

**Longitudinal Bioluminescence Imaging to Monitor Breast Tumor Growth and Axitinib Treatment Response using the Chick Chorioallantoic Membrane Model**

Sumreen Javed,^1^ Sepideh Soukhtehzari,^1^ Nazarine Fernandes,^1^ and Karla C. Williams^1*^

^1^Faculty of Pharmaceutical Sciences, The University of British Columbia, Vancouver, Canada

*Corresponding author: [karla.williams@ubc.ca](mailto:karla.williams@ubc.ca)

**Keywords:** breast cancer, chick embryo, chorioallantoic membrane, tumor growth, therapeutic response, axitinib


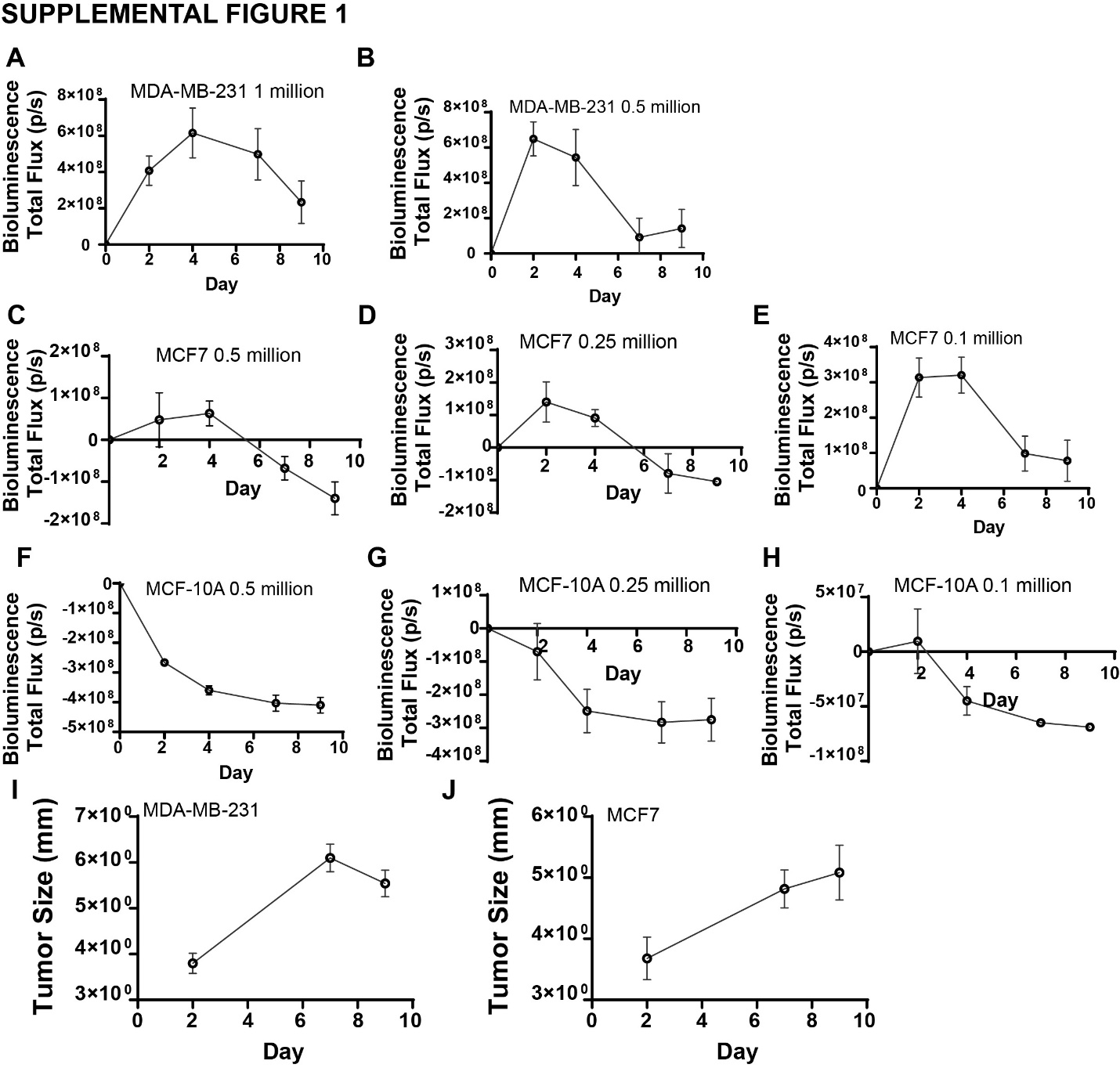


**Supplemental Figure 1. (A-H)** Growth curves of cell spheroids, as assessed by BLI, based on cell number for: MDA-MB-231 at **(A)** 10^6^ and **(B)** 5x10^5^cells/sphere, MCF7 at **(C)** 5x10^5^ **(D)** 2.5x10^5^ and **(E)** 1x10^5^ cells/sphere, and MCF-10A at **(F)** 5x10^5^ **(G)** 2.5x10^5^ **(H)** 1x10^5^cells/sphere. **(I)** Tumor spheroid growth curve of engineered MDA-MB-231luc spheroid (2.5x10^5^ cells/spheroid) on CAM post engrafting measured through light microscope. **(J).** Tumor spheroid growth curve of engineered MCF7luc spheroid (5x10^4^ cells/spheroid) on CAM post engrafting measured through light microscope.
